# Automated Method for Intracranial Aneurysm Classification Using Deep Learning

**DOI:** 10.3390/s24144556

**Published:** 2024-07-14

**Authors:** Roberta Hlavata, Patrik Kamencay, Martina Radilova, Peter Sykora, Robert Hudec

**Affiliations:** Department of Multimedia and Information-Communication Technologies, University of Zilina, 010 26 Zilina, Slovakia or oberta.vrskova@uniza.sk (R.H.); martina.radilova@feit.uniza.sk (M.R.); peter.sykora@uniza.sk (P.S.); robert.hudec@uniza.sk (R.H.)

**Keywords:** deep learning, tumor, cancer, aneurysm, neural network, 2DCNN

## Abstract

Intracranial aneurysm (IA) is now a common term closely associated with subarachnoid hemorrhage. IA is the bulging of a blood vessel caused by a weakening of its wall. This bulge can rupture and, in most cases, cause internal bleeding. In most cases, internal bleeding leads to death or other fatal consequences. Therefore, the development of an automated system for detecting IA is needed to help physicians make more accurate diagnoses. For this reason, we have focused on this problem. In this paper, we propose a 2D Convolutional Neural Network (CNN) based on a network commonly used for data classification in medicine. In addition to our proposed network, we also tested ResNet 50, ResNet 101 and ResNet 152 on a publicly available dataset. In this case, ResNet 152 achieved better results than our proposed network, but our network was significantly smaller and the classifications took significantly less time. Our proposed network achieved an overall accuracy of 98%. This result was achieved on a dataset consisting of 611 images. In addition to the mentioned networks, we also experimented with the VGG network, but it was not suitable for this type of data and achieved only 20%. We compare the results in this work with neural networks that have been verified by the scientific community, and we believe that the results obtained by us can help in the creation of an automated system for the detection of IA.

## 1. Introduction

Nowadays, there is increasing discussion about the occurrence of Subarachnoid Haemorrhage (SAH) and the term Intracranial Aneurysm (IA). These terms are closely related, as the rupture of an IA can cause SAH. IAs are brain aneurysms, meaning they occur in the brain. More precisely, aneurysms are bulges in blood vessels that can burst or leak, causing the aforementioned SAH. Currently, Intracranial Aneurysms (IAs) mostly affect people under the age of 65 and have a very high mortality and morbidity rate. Research has shown that women are more likely to develop IAs [1,2]. However, approximately 3% of healthy adults have an intracranial aneurysm [2]. The need for a correct and accurate diagnostic approach for such a dangerous condition is critical. However, diagnosis in this case is very difficult. Various approaches are used for diagnosis, such as Digital Subtraction Angiography (DSA), Computed Tomography Angiography (CTA) and Magnetic Resonance Angiography (MRA). The difference between these approaches lies in resolution and detection sensitivity, with CTA and MRA providing lower resolution than DSA [2].

In order to speed up and make the detection of IA more accurate, many approaches have been created in recent years that could help in the detection of IA. Authors of different approaches focused on machine learning and artificial neural networks. In the article [3], the authors proposed an IA-detection method using MRA recordings, where they obtained 154 files for training and 113 for validation. For semantic segmentation, they used 3D U-Net with an auxiliary classifier. The authors’ proposed method achieved an accuracy of 0.910 in internal validation and an accuracy of 0.883 in external validation. The authors of the article [4] worked with data obtained from CTA images. The authors of the approach worked with 3D images and applied the 3D convolutional neural network (3D CNN) architecture on the DeepMedic platform. They achieved a segmentation accuracy of IA from CTA of 92.3%. In the article [5], the authors used the segmentation and classification network JSCD-Net on 3D images from MRA. The authors achieved a sensitivity of 91.2%. The paper [6] dealt with the detection of IA using a two-channel ResNet classifier from CTA images. The authors of the article achieved a sensitivity of 90%. The authors in the article [7] used a convolutional neural network (CNN) for detection. On CTA images, sensitivities reached 92%. In the article [8], the authors used a multiphase fusion deep learning model with automatic phase selection on CTA and DSA images. The authors in the article achieved a recall of 94.8% and on DSA images 87.6%. The paper [9] deals with the modality-independent detection of IA in CTA, MRA and DSA using the ResU-net deep learning geometric model. The authors in this article obtained a sensitivity of 65.6%. In the article [10], the authors focused on the detection of IA from MR images. They achieved an F1 score of 0.802 using a deep neural network model. The authors of the article [11] were inspired by the creation of the U-Net network and trained the network on MRA images. The network created by the authors achieved a sensitivity of 65%. The authors of the article [12] focused on data preprocessing and then used 3D CNN networks, where they achieved a total accuracy of 83.3%.

In our previous research, we focused on MRA data obtained from the freely available dataset IntrA (3D Intracranial Aneurysm Dataset) [13]. This dataset is a reconstruction of scanned 2D MRA images of patients. The dataset contains 103 3D models of whole brain vessels. However, the authors do not publish the raw 2D MRA images due to medical ethics. We performed several experiments on this dataset and compared the results with those of the wider scientific community. First, we proposed a 2D CNN network [14], which was inspired by the PointCNN network. In this case, we achieved a classification accuracy of 97.36%. Subsequently, we tested other networks designed by us on this dataset. We designed a 3D CNN network [15], where we achieved an F1 score of 87.63%. Also, in the next case, we tried to improve the accuracy of the classification and modified both networks [16] and then on the IntrA dataset, we obtained F1 score results on the proposed 2D CNN network of 90.45% and on the 3D CNN network we achieved an F1 score of 90.97%. In our article, however, we decided to move further in the research and use another imaging modality, namely CTA, where we tested several types of neural networks on the freely available Tumor, Cancer and Aneurysm Detection Image Dataset [17]. We designed a 2D Convolutional Neural Network (2D CNN) that achieved an accuracy of 98%. Subsequently, we tested ResNet 50, ResNet 101 and ResNet 152 on the aforementioned dataset. We achieved approximate results with our proposed neural network and with ResNet152, namely 99.17%. However, our network was significantly smaller, and training and classification took less time. In this case, we tried to test VGG networks on the obtained data, but they were not sufficient for this type of problem and achieved a classification accuracy of only 20%. We believe that the achieved results can significantly help in the issue of IA detection.

## 2. Materials and Methods

In our research, we focus on the detection of aneurysms from images. Networks such as various types of Convolutional Neural Networks (CNNs), including specific architectures like ResNet and VGG, are popular for image anomaly detection. These networks are often used for the classification of medical images.

### 2.1. 2D CNN Network Architecture

**Figure 1:**
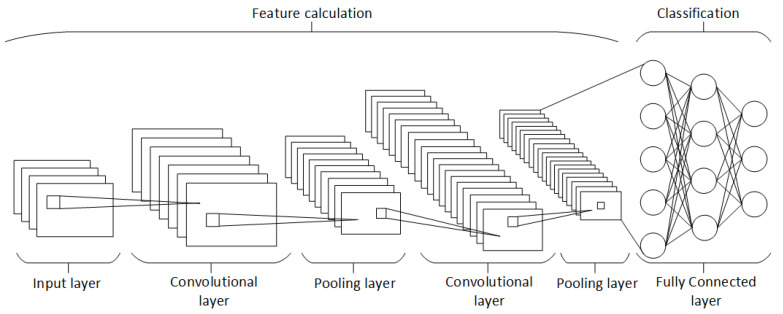
Block diagram of 2D CNN architecture [18].

The 2D Convolutional Neural Network (2D CNN) is a type of deep learning model specifically designed for processing grid-like data, such as images (see Figure 1). It consists of several key layers that work together to learn and extract features from the input data [18]:

- Input Layer;
- Convolutional Layers;
- Activation Function (ReLU);
- Pooling Layers;
- Fully Connected (Dense) Layers;
- Output Layer.

The architecture of a 2D CNN is designed to automatically and adaptively learn spatial hierarchies of features from the input data, making it highly effective for tasks involving image and video recognition [18].

### 2.2. VGG and ResNet Network Architecture

**Figure 2:**
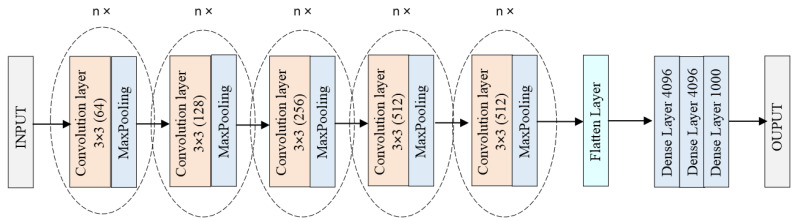
Block diagram of VGG architecture [19].

Visual Geometry Group (VGG) is a convolutional neural network (CNN) architecture proposed by researchers from Oxford University in 2014. It is known for its simplicity and effectiveness in image-recognition tasks. VGG networks come in different variations, notably VGG16 and VGG19, which consist of 16 and 19 layers, respectively. The architecture is composed of a series of convolutional layers with filters of the same size, followed by 3 fully connected layers. The ReLU activation function is used after the dense layers to introduce non-linearity and enhance learning performance (see Figure 2) [19].

**Figure 3:**
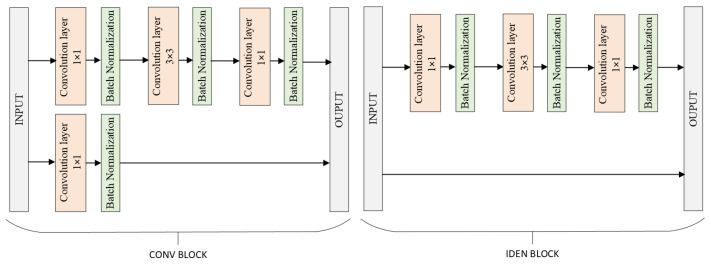
Block diagram of ResNet architecture [20].

Residual Neural Network (ResNet) is a CNN architecture introduced by Microsoft Research in 2015. It is renowned for its ability to train very deep networks by using residual learning to address the vanishing gradient problem. ResNet introduces shortcut connections, or skip connections, which allow the input of a layer to be directly added to the output of a deeper layer. This innovative approach enables the construction of networks with hundreds or even thousands of layers. The most common variants are ResNet50, ResNet101 and ResNet152, with the number indicating the depth of the network. ResNet’s architecture has significantly improved performance on various image-recognition benchmarks (see Figure 3) [20].

### 2.3. Tumor, Cancer and Aneurysm Detection Image Dataset

**Figure 4:**
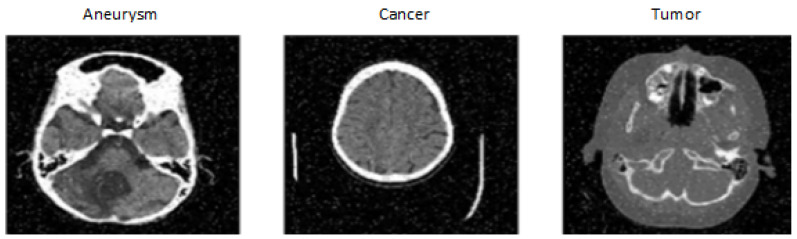
Tumor, Cancer and Aneurysm Detection Image Dataset [17].

The dataset was created by DiscoverAI [17], for the general public to train neural networks and achieve results in the detection of aneurysms, tumors and cancer. This dataset contains 611 images from CT (see Figure 4) on which the given diseases occur. CT images are resized to 640 × 640. The images in the dataset also contain significant noise up to 5% of pixels. Images with noise can complicate disease classification. The dataset consists of three classes:

- Aneurysm: contains only CT images showing different types of intracranial aneurysms.
- Tumor: contains only CT images showing different types of tumors occurring in the brain.
- Cancer: contains only CT images showing different types of cancer in the brain.

We chose this dataset because of the change in the modality of availability of CT images and the inclusion of several classes containing three different diseases. This makes classification more challenging, as a tumor in the area of a blood vessel can be mistaken for an aneurysm, and vice versa. The dataset is divided into training, testing and validation sets in the ratio 87:8:5. Another important factor in selecting this dataset was that the company Roboflow also published a model on this dataset, achieving a total validation accuracy of 98%, which we aimed to match while also speeding up the classification time [17].

### 2.4. Proposed 2D CNN Architecture

**Figure 5:**
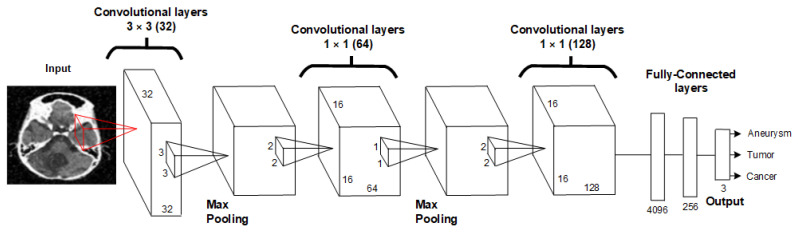
Proposed architecture of 2DCNN.

Our proposed neural network architecture is composed of 2D convolution layers. The architecture consists of three convolution layers and three MaxPooling layers. We aimed to design the 2D CNN architecture to be as simple as possible to ensure that the resulting model is minimally demanding on computing power and that classification occurs as quickly as possible. The proposed architecture is shown in Figure 5 and Table 1.

The proposed 2D CNN architecture starts with an input layer that processes 640 × 640 pixel images, followed by a series of convolutional and max-pooling layers that extract spatial hierarchies of features. The network includes three convolutional layers with increasing filter sizes and ReLU activation functions, interspersed with max-pooling layers to reduce spatial dimensions and computational load. The extracted features are then passed through two fully connected dense layers with ReLU activation and dropout regularization to mitigate overfitting. The final output layer, equipped with either a softmax or sigmoid activation function, delivers the classification result. This architecture consists of three 2D convolution layers, one dense layer, a dropout layer and a flatten layer. The 2D CNN architecture was designed to place the least possible burden on computing power while achieving the highest possible accuracy values. Hyperparameters of the 2D convolutional layers and MaxPooling are set to achieve the best results. The first 2D convolutional layer has 32 filters with a kernel size of 3 × 3 and a stride of 1, followed by MaxPooling with a window size of 2 × 2. The next 2D convolutional layer has 64 filters with a kernel size of 1 × 1 and a stride of 1, with MaxPooling layers of 2 × 2 following each convolution layer. The final 2D convolutional layer has 128 filters, a kernel size of 1 × 1 and a stride of 1. This is followed by a dropout layer with a value of 0.5, a dense layer with 512 units and finally, a flatten layer. Mathematical description of the individual steps:

1. **Input Layer**:
  $$X\in R^{640\times 640}$$
  where X is the input image with dimensions 640×640.
2. **First Convolutional Layer**:
  $$H_{1}=\mathrm{ReLU}\left(W_{1}*X+b_{1}\right)$$
  where $W_{1}$ are the filters of size 3×3, $b_{1}$ is the bias and ∗ denotes the convolution operation. The stride is 1.
3. **First Max-Pooling Layer**:
  $$P_{1}=\mathrm{MaxPooling}\left(H_{1},\;2\times 2\right)$$
  reducing the spatial dimensions by a factor of 2.
4. **Second Convolutional Layer**:
  $$H_{2}=\mathrm{ReLU}\left(W_{2}*P_{1}+b_{2}\right)$$
  where $W_{2}$ are the filters of size 1×1, and the stride is 1.
5. **Second Max-Pooling Layer**:
  $$P_{2}=\mathrm{MaxPooling}\left(H_{2},\;2\times 2\right)$$
6. **Third Convolutional Layer**:
  $$H_{3}=\mathrm{ReLU}\left(W_{3}*P_{2}+b_{3}\right)$$
  where $W_{3}$ are the filters of size 128×1×1×64, and the stride is 1.
7. **Third Max-Pooling Layer**:
  $$P_{3}=\mathrm{MaxPooling}\left(H_{3},\;2\times 2\right)$$
8. **Dropout Layer**:
  $$D=\mathrm{Dropout}(P_{3},\;p=0.5)$$
  where p=0.5 is the dropout rate.
9. **Fully Connected Dense Layer**:
  $$F=\mathrm{ReLU}(W_{4}D+b_{4})$$
  where $W_{4}$ and $b_{4}$ are the weights and biases of the dense layer, respectively.
10. **Flatten Layer**:
  $$F_{\mathrm{flat}}=\mathrm{Flatten}\left(F\right)$$
11. **Output Layer**:
  $$O=\mathrm{Softmax}(W_{5}F_{\mathrm{flat}}+b_{5})$$
  where $W_{5}$ and $b_{5}$ are the weights and biases of the output layer, respectively.

The network is typically trained using a categorical cross-entropy loss function for classification tasks. The loss function is defined as follows:

$$L=-\frac{1}{N}\sum_{i=1}^{N}\sum_{c=1}^{C}y_{i,c}\log\left({\hat{y}}_{i,c}\right)$$

where *N* is the number of samples in the batch, *C* is the number of classes, $y_{i,c}$ is the binary indicator (0 or 1) if class label *c* is the correct classification for sample *i* and ${\hat{y}}_{i,c}$ is the predicted probability of sample *i* being in class *c*. The our goal is to minimize this loss function during training to improve the accuracy of the network’s predictions. This architecture balances complexity and performance, aiming to deliver high accuracy in detecting intracranial aneurysms while maintaining computational efficiency. The network’s design allows for rapid training and inference, making it suitable for integration into clinical settings where timely decision-making is critical.

Subsequently, we used these hyperparameters to train the neural network model (see Table 2). Adamax, a variant of the Adam optimizer, was employed for training (robustness and stability in handling sparse gradients). It combines the advantages of AdaGrad and RMSProp, making it suitable for large-scale data and high-dimensional parameter spaces. A learning rate of 0.001 was chosen to balance between convergence speed and stability, ensuring that the model effectively learns from the data without overshooting the optimal parameters. This number was selected to provide sufficient training time for the network to learn the underlying patterns in the data. The number of epochs was set to 50. The momentum of 0.5 was used to help the optimizer converge more quickly and avoid local minima (this helps accelerate gradients vectors in the right directions). The logarithmic interval was set to record the interval every 10 steps, providing periodic updates and checkpoints during the training process (monitoring the performance of the model at regular intervals). The batch size was set to 16. A smaller batch size was chosen to reduce memory usage and potentially improve the generalization of the model, though it may increase the training time.

The deep learning systems were created using Python libraries such as Pytorch, and the experiment results were obtained using Nvidia CUDA libraries. These hyperparameters were carefully selected based on empirical evidence and best practices in deep learning to optimize the performance and efficiency of the proposed neural network model.

## 3. Experimental Results

This section presents the experimental results achieved on the Tumor, Cancer and Aneurysm Detection Dataset. To provide a more comprehensive comparison of the proposed architecture, we conducted a comparative analysis of experimental results obtained on these datasets from neural networks such as ResNet50, ResNet101, ResNet152 and VGG16. Furthermore, the results were compared with those obtained by the model from RoboFlow. In this study, the data set was divided into three subsets: training, test and validation. The distribution of the data was 87:8:5.

### Results

**Figure 6:**
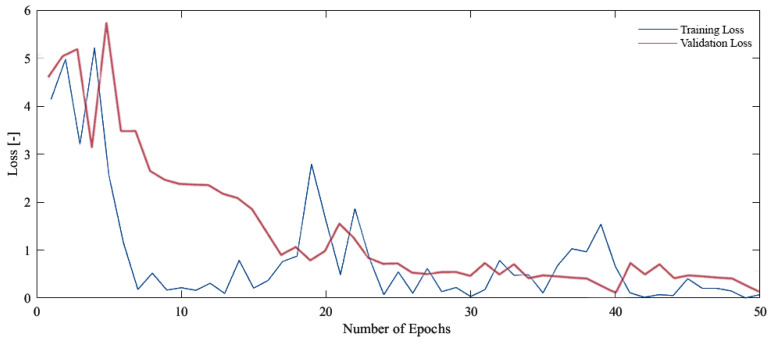
Loss function during training and validation process our proposed NN on the Tumor, Cancer and Aneurysm Detection Image Dataset.

In this study, the initial step was to classify the dataset that contained our problem of interest, namely aneurysms. For the experiments, we therefore used the Tumour, Cancer and Aneurysm Detection Image Dataset, which contained three categories containing aneurysms, tumors and cancer. The data entered the neural network in size 640 × 640, and no image pre-processing was employed. During the training phase, we monitored the decrease in the loss function and the overall training accuracy. The loss function of the proposed neural network can be seen in the graph shown in Figure 6. The graph shows that the loss function of the NN proposed by us dropped significantly during the first epochs of training and subsequently oscillated around the value of one. However, from an overall point of view, we can observe a decrease in the loss function during the entire training phase.

**Figure 7:**
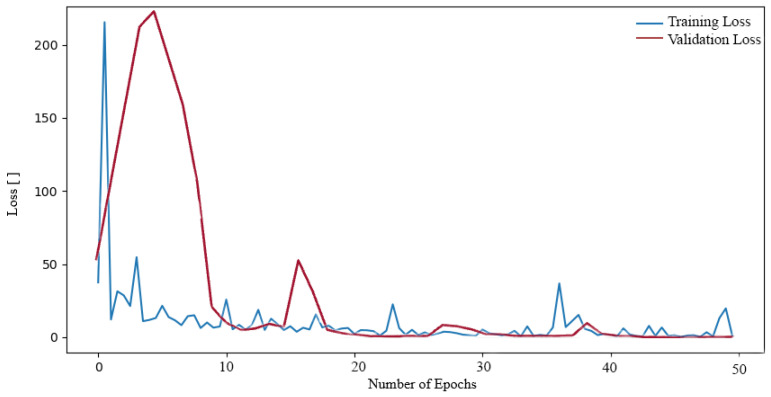
Loss function during training and validation process ResNet50 on the Tumor, Cancer and Aneurysm Detection Image Dataset.

Subsequently, we proceeded to train additional neural networks (NNs) on the aforementioned dataset. Initially, we trained the NN ResNet50. A decrease in values can also be observed in the loss function during ResNet50 training (see Figure 7). In this case, however, the decrease is more rapid at higher values of the loss function, where at the beginning it reached values of approximately 200. Nevertheless, a slight oscillation was also observed throughout the entirety of the training period, exhibiting a relatively minor amplitude when compared to the initial decline from higher values. Nevertheless, a low loss function value of 0.0603 was also attained.

**Figure 8:**
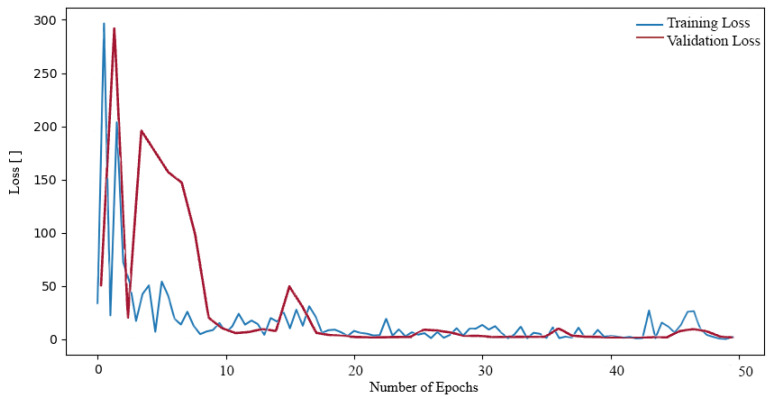
Loss function during training and validation process ResNet101 on the Tumor, Cancer and Aneurysm Detection Image Dataset.

In other instances, the ResNet101 network was subjected to further training on the aforementioned dataset. This resulted in a reduction in the loss function during the training process, as illustrated in Figure 8. In the case of the ResNet101 network, the decline process exhibited a similar pattern to that observed in the ResNet50 network. The loss function initially reached exceedingly high values, reaching as high as 300. In the subsequent epochs, the loss function value decreased to a lower number, oscillating around the value of 1. The resulting loss function value was 0.0504.

**Figure 9:**
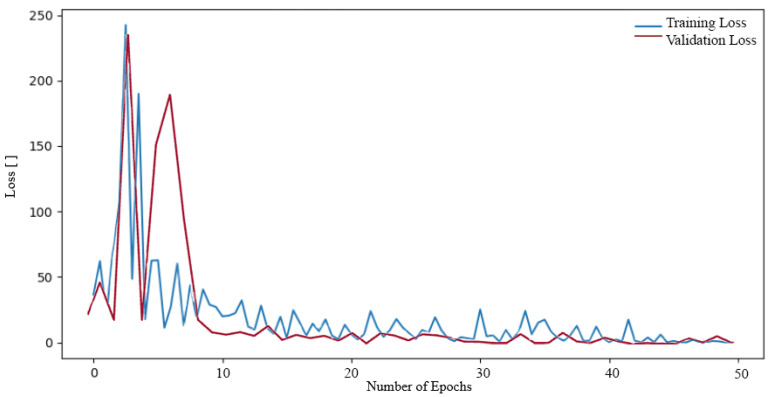
Loss function during training and validation process ResNet152 on the Tumor, Cancer and Aneurysm Detection Image Dataset.

Finally, the ResNet152 network was trained on the dataset. The decrease in loss function during training was monitored (see Figure 9). The plot of the training was similar to that of the previous networks, with the loss function reaching a high value of around 250 in the first epochs. Subsequently, in the subsequent epoch, the loss function exhibited a decline to values approaching 1. It oscillated at this value until it ultimately reached the final loss function during training, which was 0.0706.

On the other hand, even though we also trained the VGG16 network on the available dataset, its loss function reached significantly high values during the entire training and was not able to train on the data. Also, even these high values of the loss function did not decrease at all, rather they oscillated without a decreasing tendency. Based on this information, we did not work with the networks any further. In the Table 3, we can see an overview of metrics used during training, such as loss function and accuracy, for all networks that we trained on the dataset. At the same time, information about the loss function and accuracy test metrics can also be seen on the panel. Here we observe that very good results were achieved in all cases of the resNet network. However, our network and the ResNet152 network achieved the best results both during training and during testing. In our case, the lice network is significantly simpler and less computationally demanding than the ResNet152 network. The network designed by us achieved a train accuracy of 97.52% and a test accuracy of 98.01%. At the same time, the network designed by us achieved a train loss function of 0.0538 and a test loss function of 0.0360. The ResNet152 network achieved a train accuracy of 99.17% and a test accuracy of 98.00%. And at the same time, ResNet152 achieved a train loss function of 0.0706 and a test loss function of 0.0770.

**Table 3:** The accuracy and loss function of the model over 50 epochs, during both training and testing phases using the Tumor, Cancer and Aneurysm Detection Image Dataset.

Evaluation Metrics	Proposed 2D CNN	Resnet152	ResNet101	ResNet50	VGG16
Train loss	0.0538	0.0706	0.0504	0.0603	35.6563
Train accuracy	97.52%	99.17%	96.49%	94.42%	33.26%
Test loss	0.0360	0.0770	0.0514	0.0790	68.0790
Test accuracy	98.01%	98.00%	94.00%	92.00%	20.02%

**Figure 10:**
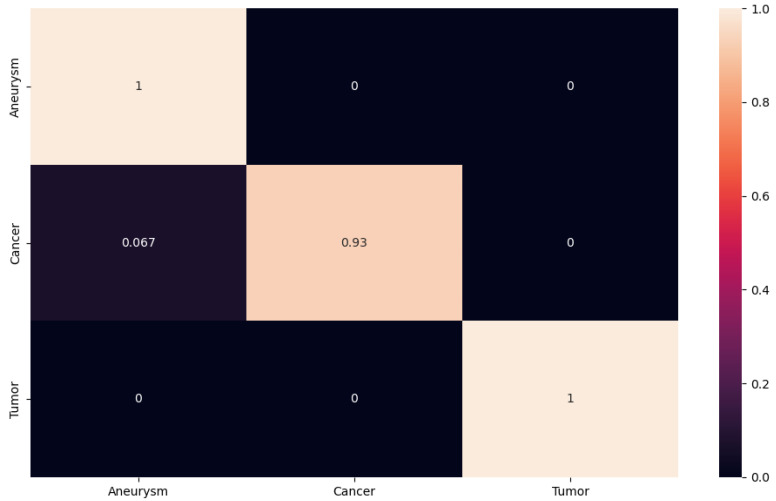
The confusion matrix for proposed 2D CNN.

For better presentation of the test results, we decided to generate the confusion matrix for our proposed network, as well as for the ResNet50, ResNet101 and ResNet152 networks. The resulting confusion matrix for our results was generated using the PyTorch library. In the matrix, we see the accuracy value for each class. The closer the value is to one, the higher the accuracy; the closer it is to zero, the less accurate the classification. The obtained experimental results using the proposed 2D CNN are shown in Figure 10. We can observe that the proposed 2D CNN had no problem with the classification of the aneurysm and tumor classes. However, it made an error when classifying the cancer class, where it misclassified some images into the aneurysm class with an accuracy of 0.067. This error rate may have occurred because some cancerous foci may appear from certain angles like an aneurysm on a blood vessel.

**Figure 11:**
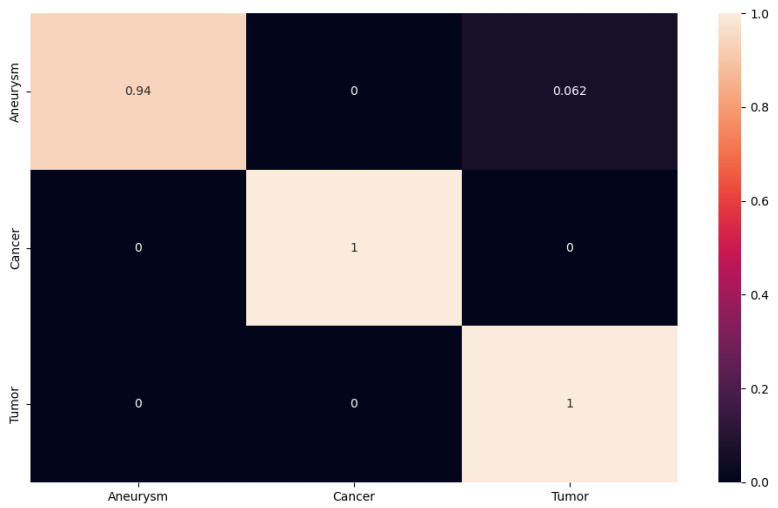
The confusion matrix for ResNet152.

In another case, we can follow the confusion matrix on ResNet152, which achieved approximately the same results as the network suggested to us (see Figure 11). In this case, however, we observe that ResNet152 had no problem with tumor and cancer classification. The network encountered an aneurysm class error rate. In our case, the aneurysm class is the most important, as our goal is to design a NN that will classify and detect aneurysms from different modalities as accurately as possible. In this case, the network misclassified with an accuracy of 0.062 images from the aneurysm class to the tumor class.

**Figure 12:**
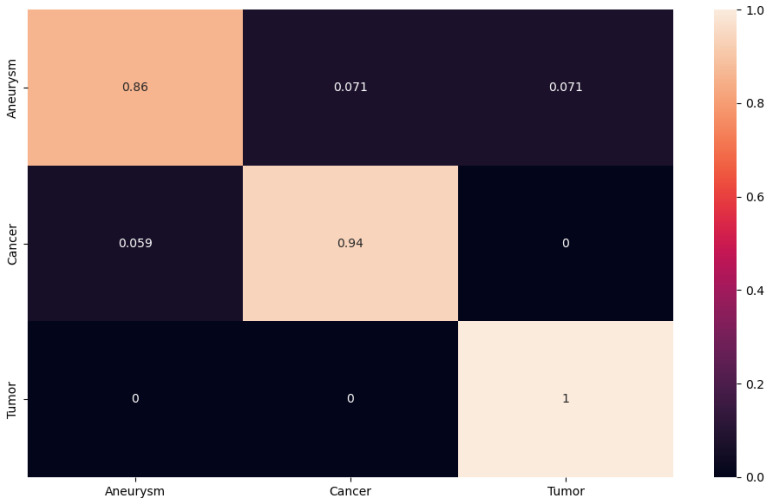
The confusion matrix for ResNet101.

**Figure 13:**
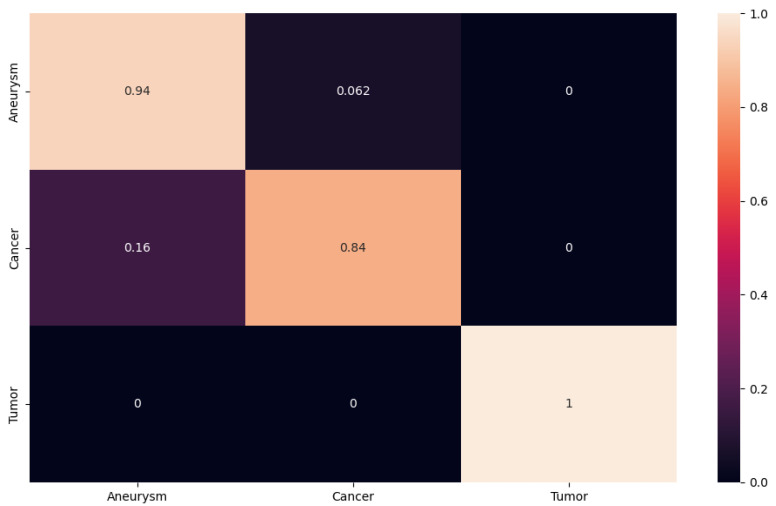
The confusion matrix for ResNet50.

For an overview of the achieved results, we also processed the confusion matrix for the ResNet101 and ResNet50 networks (see Figure 12 and Figure 13). In this case, both networks achieved worse results than our proposed NN and the ResNet152 network. Worse achieved results can be observed on the confusion matrix. In the confusion matrix for the ResNet101 network, we can see that the network had a significant problem with the classification of aneurysm and cancer classes, where it was wrong in both cases. For the first aneurysm class, it misclassified the images into the cancer class with an accuracy of 0.071 and at the same time, with an accuracy of 0.071, it misclassified the images into the tumor class. In the case of the cancer class, the network made a mistake only when classifying it into the aneurysm class with an accuracy of 0.059. However, from this confusion matrix, we can observe that the network had the biggest problem with the classification of the aneurysm class. With the confusion matrix calculated from the resulting data after testing from the ResNet50 network, we see a significant error rate as well as the previous network for the aneurysm class. However, we also see a fairly high classification error rate in the cancer class. In this case, the network misclassified images with an accuracy of 0.062 into the cancer class instead of the aneurysm class. Subsequently, in the cancer class, the network misclassified with an accuracy of 0.16 into the aneurysm class in the cancer class. Here we see that even though the network had an overall classification accuracy of 92% after testing, it still performed worse within the main class and had a problem with the classification of images containing aneurysms.

In order to facilitate the analysis of the results, evaluation metrics such as F1 score, precision and recall were computed for each trained network, including ResNet50, ResNet101, ResNet152, VGG16 and proposed 2D CNN. A comprehensive overview of these metrics for all networks can be found in Table 4. The metrics demonstrated that the VGG16 network encountered difficulties in training effectively with the available data. Nevertheless, both the ResNet series and our proposed network demonstrated remarkable performance in classifying the dataset.

Upon completion of our work, we conducted a comparative analysis of the results obtained on our proposed network with those achieved by the freely available ResNet and VGG networks. Additionally, we compared the results with those obtained by the finished model created for the classification of the aforementioned data. This model was created and shared on the RoboFlow domain, where it achieved 98% accuracy after testing neural networks. A comparison of the results obtained after testing can be seen in Table 5. As illustrated in the table, the network designed by us exhibited a throughput that was approximately one-tenth of a unit higher than that of all other networks. However, the network proposed by us was relatively simple, comprising only three convolutional layers and fully connected layers. This simplicity primarily influenced the computing power and the speed of classification.

In this case, we can posit that we have proposed a model for classification that is both capable and applicable in practice, thereby simplifying the work of doctors in the detection of aneurysms. The dataset encompasses three distinct disease categories, in addition to aneurysms, tumors and cancer. Additionally, we created a table (see Table 6) to facilitate a comparative analysis of the classification results, with a particular focus on the aneurysm class. Our objective was to developed a neural network (NN) that can accurately classify aneurysms across different modalities, thereby enabling the prediction of the adverse outcomes associated with aneurysms.

**Table 5:** Accuracy comparison of the proposed architecture with various neural network.

Neural Networks	Accuracy (%)
Proposed 2D CNN	98.01
VGG16 [19]	20.02
Resnet50 [20]	92.00
Resnet101 [20]	94.00
Resnet152 [20]	98.00
RoboFlow model NN [17]	98.00

**Table 6:** Comparison of the accuracy of the proposed architecture with different neural networks.

Neural Networks	Accuracy (%)
Proposed 2D CNN	99.99
VGG16 [19]	11.11
Resnet50 [20]	94.00
Resnet101 [20]	86.00
Resnet152 [20]	94.00
RoboFlow model NN [17]	94.00

Table 6 illustrates that the network proposed by us in the overall classification yielded results that were equivalent to those achieved by the RoboFlow and ResNet152 models. However, the network proposed by us is primarily tailored to the aneurysm class. Within the aneurysm class, the neural network (NN) achieved a classification accuracy of 99.99%.

## 4. Conclusions and Future Work

The main objective of this article was to present a straightforward neural network-classification approach that can be applied to not only aneurysms but also cancer and tumor. The network’s primary architectural component was a series of 2D convolution layers. The network was trained and tested on the Tumor, Cancer and Aneurysm Detection Image Dataset, which is freely available. The input CT images were input into the neural networks in a size of 640 × 640. In order to confirm the relevance of our results, we conducted a series of tests. In order to assess the efficacy of our approach, we conducted a comparative analysis with the ResNet50, ResNet101 and ResNet15 networks, as well as the VGG16 network, which was applied to the dataset. As the dataset is freely available in the RoboFlow community, we also compared the results with their proposed 2D convolutional neural network (CNN) model. The results demonstrated the efficacy of the proposed 2D CNN neural network in classifying CT images containing aneurysms, tumors and cancer. Training on the Tumor, Cancer and Aneurysm Detection Image Dataset resulted in a reduction of the loss function to 0.0360 and an increase in accuracy to 98%. To facilitate a comparative analysis, the results were also evaluated in conjunction with other neural network models. The ResNet50 model exhibited a loss function of 0.0790, while the ResNet101 model demonstrated a loss function of 0.0514 and the ResNet152 model exhibited a loss function of 0.0770. The RoboFlow network model exhibited a loss function of 0.0250. The VGG16 network model was unable to be trained on the data set. The confusion matrices for the majority of the models demonstrated that the classification process was successful, with minimal errors in the average of each class. In addition to the aforementioned VGG16 network, The overall test accuracy of the network was 98%, with precision and recall values of 97.76% and 97.91%, respectively. The F1 score was 97.76%. The results demonstrated that the proposed 2D CNN neural network is capable of accurately classifying CT images containing aneurysms, with minimal errors. The proposed architecture demonstrated comparable performance to other existing networks designed by models and architectures. However, the advantage of our network is that it is compact and does not necessitate high-performance computing.

Nevertheless, our objective is to persist in our endeavors and enhance the efficacy of the classification of intracranial aneurysms. Additionally, the objective is to construct a network that yields optimal outcomes with minimal computational burden and a high classification speed. The accurate classification of intracranial aneurysms using neural networks has the potential to significantly enhance their detection, thereby reducing the risk of fatal consequences associated with aneurysms. To provide a more comprehensive evaluation of the performance of our network, we conducted a comparative analysis with the previously mentioned neural network architectures and trained models, including ResNet approaches 50, 101 and 152, and the RoboFlow model. This paper primarily addresses the recognition and classification of intracranial aneurysms, which have a significant impact on the scientific and medical communities as well as the general public. The proposed 2D CNN architecture has a wide range of applications in numerous areas where image classification is required. In conclusion, the proposed 2D CNN is a straightforward, low-performance and highly effective deep learning architecture.

In future work, we intend to investigate the inclusion of additional datasets pertaining to aneurysms and to evaluate the efficacy of multiple approaches for enhancing the performance of the proposed model. We also intend to explore the potential of transfer learning techniques for adapting the model to diverse domains and environments. Finally, we will examine the feasibility of deploying the model on real-time monitoring devices, which could assist physicians in detecting and preventing the fatal consequences that aneurysms can cause.

## Figures and Tables

**Table 1:** Description of the layers of the proposed 2DCNN.

Layers	Description of Layers
Conv2D_1	32 filters with dimensions 3 × 3, the output is a feature map with dimensions 32 × 32 × 32
MaxPooling_1	Filter size 2 × 2
Conv2D_2	64 filters with dimensions 1 × 1, the output is a feature map with dimensions 16 × 16 × 64
MaxPooling_2	Filter size 2 × 2
Conv2D_3	128 filters with dimensions 1×1, the output is a feature map with dimensions 16 × 16 × 128
MaxPooling_3	Filter size 2 × 2
Dropout_1	50% neuron shutdown
Flatten_1	4096 neurons
Dense_1	256 neurons
Dropout_3	50% neuron shutdown
Dense_2	3 neurons

**Table 2:** Hyperparameters used during training and evaluation of the proposed 2D CNN.

Hyperparameter	Value
Optimization Algorithm	Adamax
Learning Rate	0.001
Training Epochs	50
Momentum	0.5
Logarithmic Interval	10
Batch Size	16

**Table 4:** The evaluation metrics of the proposed neural network with various neural network using the Tumor, Cancer and Aneurysm Detection Image Dataset.

Evaluation Metrics	Proposed 2D CNN	Resnet152	ResNet101	ResNet52	VGG16
Precision (P)	97.76%	97.94%	93.31%	92.60%	33.33%
Recall (R)	97.91%	98.05%	93.31%	92.86%	11.11%
F1 score (F1)	97.76%	97.93%	93.21%	92.59%	16.67%

## Data Availability

The data presented in this study are available on request from the corresponding author. This is according to the laboratory rules.

## Abbreviations

The following abbreviations are used in this manuscript:

2D	Two-Dimensional
2D CNN	Two-Dimensional Convolutional Neural Network
AI	Artificial Intelligence
CNN	Convolutional Neural Network
ConvLSTM	Convolutional Long Short-Term Memory

CT	Computed Tomography
CTA	Computed Tomography Angiography
DICOM	Digital Imaging and Communications in Medicine
DNN	Deep Neural Network
IA	Intracranial Aneurysm
MRI	Magnetic Resonance Imaging
MRA	Magnetic Resonance Angiography
NN	Neural Network
